# Supplementary material for: Development of Sense of Coherence Stability in the AGORA Healthy Ageing Study
Source: Int J Environ Res Public Health. 2022 Oct 30;19(21):14190. doi: 10.3390/ijerph192114190 (PMC9657695; doi:10.3390/ijerph192114190)
Supplement: Supplementary file 1 [file ijerph-19-14190-s001.zip › ijerph-1924553-supplementary.pdf]

Supplementary Table S1. Negative life events among the participants in the 12 months prior the follow up (2010, 2013).

SOC	Severe illness of oneself			
	2008-2010		2010-2013	
	Yes	No	Yes	No
-3	1	10	4	12
-2	3	11	1	16
-1	2	57	11	63
0	26	215	25	215
1	6	35	8	36
2	2	29	2	17
3	4	6	2	11

SOC	Severe illness of partner or family member			
	2008-2010		2010- 2013	
	Yes	No	Yes	No
-3	1	10	4	13
-2	3	12	2	14
-1	7	57	12	60
0	34	218	25	214
1	9	35	4	39
2	3	29	2	18
3	3	27	1	11

	Admission of the partner in nursing home or in home for the elderly			
SOC	2008-2010		2010-2013	
	Yes	No	Yes	No
-3	0	10	0	16
-2	0	15	0	16
-1	1	62	1	70
0	5	243	3	235
1	1	44	0	43
2	1	32	1	19
3	0	30	0	11

	Death of the partner			
SOC	2008-2010		2010- 2013	
	Yes	No	Yes	No
-3	1	10	1	15
-2	0	15	1	16
-1	2	64	5	66
0	6	241	5	232
1	0	46	1	42
2	0	32	2	18
3	3	28	0	11

	Death of a close relative different from partner			
SOC	2008-2010		2010- 2013	
	Yes	No	Yes	No
-3	3	9	4	12
-2	2	13	1	14
-1	10	53	11	64
0	43	206	38	206
1	9	37	4	38
2	6	26	2	18
3	6	25	1	9

	Important degradation of financial situation			
SOC	2008-2010		2010- 2013	
	Yes	No	Yes	No
-3	1	12	1	15
-2	3	12	2	15
-1	2	63	3	71
0	11	254	27	211
1	4	42	3	38
2	1	32	1	18
3	3	30	3	10

	Divorce or break up with partner			
**SOC**	**2008-2010**		**2010- 2013**	
	**Yes**	**No**	**Yes**	**No**
**-3**	1	11	0	16
**-2**	0	15	0	17
**-1**	0	64	0	74
**0**	0	250	0	237
**1**	0	46	0	43
**2**	0	33	0	19
**3**	0	40	0	11

	Flaming row or severe conflict			
**SOC**	**2008-2010**		**2010- 2013**	
	**Yes**	**No**	**Yes**	**No**
**-3**	0	13	1	15
**-2**	0	14	1	16
**-1**	1	64	2	73
**0**	3	259	7	233
**1**	0	46	0	43
**2**	0	33	0	19
**3**	2	31	0	11

	Other radical events			
**SOC**	**2008-2010**		**2010-2013**	
	**Yes**	**No**	**Yes**	**No**
**-3**	1	10	1	12
**-2**	1	12	2	12
**-1**	0	60	6	64
**0**	11	235	9	213
**1**	2	41	1	41
**2**	2	28	0	18
**3**	1	28	0	10

All values are given as frequencies. Values from -3 to 3 indicates the percentile membership for each participant.
